# Coagulation abnormalities in pediatric anemia clinical and laboratory correlates for risk stratification and risk assessment

**DOI:** 10.1097/MS9.0000000000004585

**Published:** 2025-12-16

**Authors:** Emmanuel Ifeanyi Obeagu

**Affiliations:** a Department of Biomedical and Laboratory Science, Africa University, Zimbabwe; b The Department of Molecular Medicine and Haematology, School of Pathology, Faculty of Health Sciences, University of the Witwatersrand, Johannesburg, South Africa

**Keywords:** anemia, coagulation abnormalities, hemostasis, pediatrics, risk assessment

## Abstract

Anemia affects an estimated 40% of children globally, with iron deficiency, chronic disease, hemoglobinopathies, and acute blood loss as leading causes. In addition to impairing oxygen delivery, anemia can disrupt normal hemostasis, leading to a range of coagulation abnormalities, including thrombocytopenia, reactive thrombocytosis, platelet dysfunction, altered coagulation factor levels, and abnormal fibrinolysis. The type and severity of these coagulation changes vary according to the underlying anemia etiology, influencing the risk of bleeding or thrombotic complications. This narrative review synthesizes current clinical and laboratory evidence on coagulation disturbances in pediatric anemia, highlighting their prevalence, pathophysiologic mechanisms, and clinical manifestations. Emphasis is placed on practical strategies for risk assessment, integrating standard laboratory evaluations – complete blood count, coagulation panel, and platelet function assays – with clinical features to identify children at highest risk for adverse outcomes. The review also discusses implications for individualized management, including etiology-directed therapy, supportive hemostatic measures, and caregiver education to improve safety and outcomes. Future research priorities include the development of standardized risk stratification tools and evidence-based pediatric management guidelines to optimize care in this vulnerable population. This review underscores the need for early coagulation monitoring in anemic children and recommends integrating clinical features with laboratory markers to enhance risk stratification and guide timely, individualized management.

## Introduction

Anemia remains one of the most prevalent hematological disorders among children worldwide, affecting both developed and developing countries^[1,2]^. It is particularly common in children under the age of 5 years, with iron deficiency, chronic infections, inherited hemoglobinopathies, and nutritional insufficiencies being leading causes. While the direct consequences of anemia – such as fatigue, impaired growth, and developmental delays – are widely recognized, its potential to alter coagulation dynamics and predispose to thrombotic or hemorrhagic events has garnered increasing clinical attention^[3,4]^. The hemostatic system in children is a dynamic and evolving network that differs from that of adults in both composition and function. In pediatric patients, especially those with comorbid conditions like anemia, this balance can be easily disrupted.

Emerging data suggest that anemia may significantly influence coagulation pathways through mechanisms such as altered platelet function, endothelial activation, and changes in blood viscosity. These changes can present subtly in laboratory findings or manifest clinically as bleeding diathesis or thrombosis, depending on the underlying type of anemia^[5]^. Iron deficiency anemia (IDA), the most common form of anemia in children, is frequently associated with thrombocytosis and changes in platelet reactivity^[6,7]^. Studies suggest that iron plays a regulatory role in megakaryopoiesis, and its deficiency may lead to exaggerated platelet production^[8,9]^. This reactive thrombocytosis, though often benign, has been implicated in thrombotic events, especially in the presence of additional risk factors such as infection, dehydration, or inflammation^[10]^. Therefore, IDA presents a paradoxical risk profile that merits close monitoring.

Anemia is one of the most prevalent hematologic conditions in childhood and continues to represent a pressing global health concern. According to the World Health Organization’s 2023 estimates, approximately 40% of children under 5 years and 37% of school-aged children worldwide are anemic, with the highest burden in low- and middle-income countries. The clinical significance of anemia in pediatrics extends beyond reduced oxygen-carrying capacity, as emerging evidence highlights its interplay with hemostatic balance and coagulation dysfunction^[5–7]^. Mechanistically, different anemia subtypes contribute to coagulation abnormalities through distinct yet interconnected pathways. In iron deficiency anemia, hypoxia-inducible factor (HIF) pathways are activated, leading to increased erythropoietin production and modulation of vascular endothelial function, which may alter platelet reactivity^[8]^. Hemolytic anemias such as sickle cell disease and thalassemia introduce additional complexity through chronic oxidative stress, free hemoglobin release, and nitric oxide depletion, each of which disturbs endothelial integrity and coagulation cascades. In bone marrow failure syndromes, cytopenias and compensatory inflammatory responses contribute to fragile hemostatic control. Collectively, these mechanisms underscore that pediatric anemia is not merely a disorder of red cell insufficiency but also a contributor to broader coagulation dysregulation, warranting careful clinical and laboratory risk assessment^[9,10]^.

Conversely, hemolytic anemias such as sickle cell disease (SCD) and β-thalassemia introduce a distinct spectrum of coagulation disturbances. Chronic hemolysis, vascular occlusion, and oxidative stress in these conditions contribute to a sustained prothrombotic state. Children with SCD, for example, exhibit elevated levels of procoagulant microparticles, D-dimers, and markers of endothelial dysfunction. These laboratory abnormalities are not merely incidental but have been correlated with clinical events such as stroke, pulmonary embolism, and deep vein thrombosis, underscoring the need for proactive risk assessment^[11–13]^. Bone marrow failure syndromes and malignancies that result in pancytopenia also present unique coagulation challenges in pediatric anemia^[14]^. In these contexts, thrombocytopenia is a common finding, leading to bleeding tendencies and prolonged coagulation times. Additionally, infections, chemotherapy, and systemic inflammation can further impair coagulation factor synthesis, compounding the bleeding risk.

The aim of this narrative review is to synthesize current evidence on the complex relationship between anemia and coagulation abnormalities in pediatric patients, with particular emphasis on clinical manifestations and laboratory markers that can inform risk assessment. By integrating pathophysiological insights with practical diagnostic considerations, this review seeks to identify correlates that may serve as early indicators of bleeding or thrombotic complications. In doing so, the article not only consolidates what is known but also bridges gaps in existing literature, offering clinicians a more structured framework for evaluating and managing coagulation risks in children with anemia. This approach is intended to enhance clinical decision-making, improve patient outcomes, and provide a foundation for future research on standardized risk assessment strategies in this vulnerable population.

## Aim

This review aims to synthesize current evidence on coagulation abnormalities in pediatric patients with anemia, highlighting both clinical manifestations and laboratory correlates that can aid in risk stratification. By bridging pathophysiological mechanisms with practical diagnostic considerations, the review seeks to inform clinical practice and guide future research on improving outcomes in this population.

## Methods

**Figure 1.**
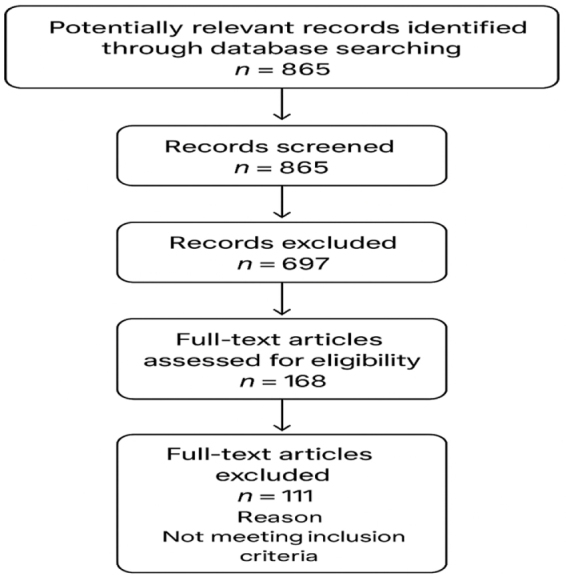
PRISMA FLOW CHART.

This narrative review was conducted following PRISMA guidelines for transparency in literature selection and reporting (Fig. 1). A comprehensive search of PubMed, Scopus, Web of Science, and Google Scholar was performed for studies published between 2000 and 2024, using combinations of the following keywords and MeSH terms: *pediatric anemia, childhood anemia, coagulation abnormalities, hemostasis, thrombosis, bleeding risk, coagulation markers, D-dimer, PT/INR, aPTT, fibrinogen, platelet indices*, and *risk stratification*. Boolean operators (AND/OR) were applied to refine the search.

All study types – observational studies, clinical trials, case series with ≥10 participants, and systematic reviews providing primary data – were eligible for inclusion if they examined coagulation parameters or hemostatic alterations in children aged 0–18 years with any form of anemia. Studies were excluded if they: (1) focused exclusively on adults; (2) evaluated coagulation markers unrelated to anemia; (3) lacked extractable clinical or laboratory data; or (4) were conference abstracts, editorials, or non–English-language publications without full-text availability.

Two reviewers independently screened the titles and abstracts of 865 identified records, followed by full-text assessment of 168 articles. Discrepancies were resolved by consensus. Ultimately, 57 studies met the inclusion criteria and were incorporated into the analysis. Data were extracted on anemia type (e.g., iron deficiency, hemolytic anemia, sickle cell disease, thalassemia, mixed etiologies), coagulation profiles (PT, aPTT, INR, fibrinogen, D-dimer), platelet indices (PLT, MPV, PDW), and reported clinical outcomes including bleeding events, thrombotic complications, and risk-stratification measures. The synthesis emphasized identifying common hemostatic alterations across anemia subtypes, evaluating diagnostic utility, and determining their relevance for clinical risk stratification.

To ensure methodological rigor, the quality of included studies was appraised using the Newcastle–Ottawa Scale for observational studies and appropriate criteria for clinical trials. Findings were narratively integrated to highlight consistent patterns, mechanistic insights, false-positive/false-negative risks, and practical applications for improving coagulation-based risk assessment in pediatric anemia.

## Limitations

While this narrative review provides a comprehensive synthesis of clinical and laboratory correlates of coagulation abnormalities in pediatric anemia, several limitations should be acknowledged. First, the included studies vary widely in design, sample size, and population characteristics, which may limit the generalizability of some findings. Many reports are observational or retrospective, increasing the potential for selection and reporting bias. Second, laboratory assessments of coagulation in children present inherent challenges. Reference ranges for hemoglobin, platelet counts, and coagulation parameters differ by age, sex, and developmental stage, which can affect interpretation. Specialized tests such as platelet function assays, thrombin generation, or D-dimer measurements may not be uniformly available, and variability in assay methodology can contribute to inconsistent results. False positives or negatives may occur, particularly in mild anemia or in the presence of concurrent illness or inflammation.

Third, data on the prevalence and clinical significance of coagulation abnormalities are limited for certain anemia subtypes, including megaloblastic anemia and anemia of chronic disease, particularly in low-resource settings. This limits precise risk stratification and the development of standardized management guidelines. As a narrative review, this study does not include formal meta-analysis or quantitative synthesis, and the evidence quality was assessed qualitatively. Nevertheless, by integrating clinical features, laboratory correlates, and pathophysiologic mechanisms, this review provides a practical framework for risk assessment and highlights priorities for future research in pediatric hematology.

## Types of anemia and their coagulation correlates

### Iron deficiency anemia

Iron deficiency anemia (IDA) is the most common form of anemia in children worldwide and is associated with distinctive alterations in hemostasis. Beyond its hematologic manifestations, IDA frequently induces a state of reactive thrombocytosis, which may increase thrombotic risk. Platelets in iron deficiency often display enhanced reactivity, with studies demonstrating heightened aggregation responses to agonists. Clinically, this can manifest as paradoxical risks: while some children present with mucosal bleeding or easy bruising, others are at risk of thrombotic complications such as cerebral venous sinus thrombosis. Laboratory findings typically reveal elevated platelet counts and increased mean platelet volume, both of which serve as correlates of hemostatic imbalance in IDA^[15]^.

### Hemolytic anemias

Hemolytic disorders, particularly SCD and thalassemia, represent a second category where coagulation abnormalities are pronounced. In SCD, chronic hemolysis releases free hemoglobin and microparticles into circulation, promoting oxidative stress, nitric oxide depletion, and endothelial activation – all of which contribute to a hypercoagulable state. Thrombotic complications, including pediatric stroke and venous thrombosis, are well-documented in this group. In thalassemia, repeated transfusions and iron overload exacerbate oxidative injury, while splenectomy further elevates thrombotic risk. Laboratory markers in hemolytic anemias often include elevated D-dimer, shortened clotting times due to increased thrombin generation, and abnormal platelet function assays. Clinically, these children may present with vaso-occlusive events, ischemic stroke, or thromboembolic disease^[16]^.

### Bone marrow failure syndromes

Bone marrow failure anemias, such as aplastic anemia and Fanconi anemia, exhibit a different profile of coagulation abnormalities, primarily dominated by bleeding tendencies. The reduced marrow reserve results in thrombocytopenia, impaired platelet function, and decreased synthesis of clotting factors, especially in the setting of hepatic involvement. These children are often prone to mucocutaneous bleeding, prolonged bleeding after minor trauma, and, in severe cases, disseminated intravascular coagulation (DIC). Laboratory findings typically demonstrate prolonged prothrombin time (PT) and activated partial thromboplastin time (aPTT), low fibrinogen levels, and reduced platelet counts, all of which reflect impaired clot formation^[17]^.

### Anemia of chronic disease

In children with chronic infections, autoimmune conditions, or malignancy-related anemia, coagulation abnormalities emerge as part of systemic inflammatory dysregulation. Elevated inflammatory cytokines enhance tissue factor express ion and activate coagulation cascades, predisposing patients to thromboinflammatory complications. Laboratory markers often show elevated fibrinogen, increased D-dimer, and subtle platelet activation, while clinical manifestations range from low-grade bleeding to thrombotic complications in severely ill children^[9]^.

## Pathophysiological interactions between anemia and hemostasis

The relationship between anemia and coagulation is increasingly recognized as multifaceted, involving overlapping mechanisms of vascular biology, platelet function, and inflammatory signaling. Hypoxia in anemic states activates hypoxia-inducible factors (HIFs), which in turn upregulate erythropoietin (EPO) and downstream mediators that influence megakaryopoiesis and platelet activation. Recent evidence highlights a crosstalk between EPO and thrombopoietin (TPO), suggesting that compensatory erythropoiesis may inadvertently enhance platelet production and function, thereby altering thrombotic risk^[15–17]^. In iron deficiency anemia, reduced hemoglobin availability and microcytosis disturb blood rheology, leading to shear stress–induced release of von Willebrand factor (vWF) from endothelial cells. Elevated plasma vWF, alongside increased soluble P-selectin as a marker of platelet and endothelial activation, reflects a prothrombotic milieu^[9,17]^. In hemolytic anemias such as sickle cell disease and thalassemia, intravascular hemolysis releases free hemoglobin and heme, scavenging nitric oxide and promoting endothelial dysfunction. This environment fosters neutrophil extracellular trap (NET) formation, or NETosis, which not only propagates inflammation but also provides a scaffold for thrombus development through interaction with fibrin and platelets^[18–20]^.

**Figure 2.**
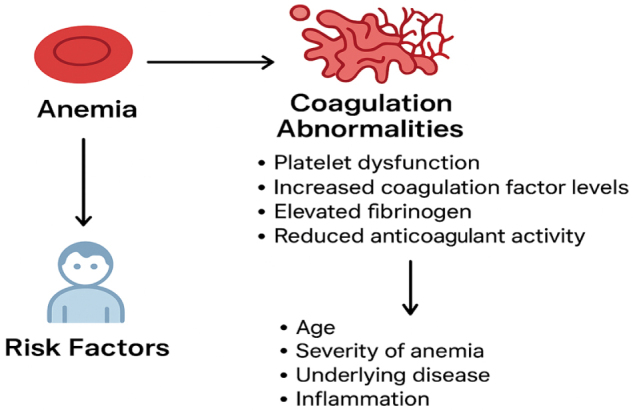
The pathophysiological interplay between anemia, coagulation abnormalities, and risk factors in pediatric patients.

Inflammation further exacerbates hemostatic imbalance. Inflammasome activation, particularly NLRP3, drives the release of interleukin (IL)-1β and IL-18, amplifying endothelial activation and tissue factor expression. Cytokines such as IL-6 and tumor necrosis factor-alpha (TNF-α) enhance hepatic synthesis of fibrinogen and acute-phase proteins, skewing the coagulation system toward hypercoagulability. In bone marrow failure syndromes and anemia of chronic disease, these inflammatory mediators, combined with quantitative cytopenias, contribute to fragile hemostatic regulation and variable risks of bleeding or thrombosis (Fig. 2)^[21,22]^.

## Clinical manifestations and risk profiles

**Figure 3.**
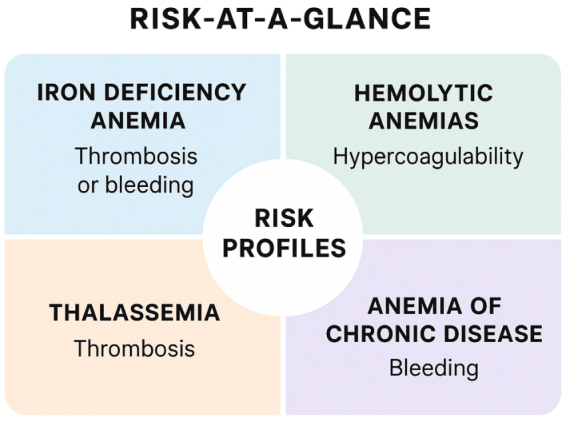
Risk-at-a glance.

In children with iron deficiency anemia, coagulation abnormalities often present as subtle yet clinically significant changes. Common manifestations include mucosal bleeding, epistaxis, and easy bruising. Paradoxically, IDA can also be associated with a modest but noteworthy risk of thrombosis, particularly cerebral venous sinus thrombosis. Observational studies suggest that thrombotic events occur in approximately 0.5–1% of pediatric patients with severe iron deficiency, highlighting the importance of careful monitoring. Laboratory findings typically reveal reactive thrombocytosis, increased mean platelet volume, and variable alterations in clotting times, which collectively help define the patient’s coagulation risk profile^[23–26]^. Hemolytic anemias, including SCD and thalassemia, exhibit pronounced hypercoagulable states. In SCD, chronic hemolysis releases free hemoglobin and microparticles, leading to endothelial dysfunction and activation of the coagulation cascade. Pediatric stroke is a particularly severe complication, with incidence estimates ranging from 5% to 11% by 18 years of age, making it one of the leading causes of morbidity in this population. Other complications include vaso-occlusive crises, silent cerebral infarcts, and venous thromboembolism. In thalassemia, thrombotic risk is heightened in transfusion-dependent patients and those post-splenectomies, due to iron overload and endothelial activation. Laboratory markers often show elevated D-dimer, shortened clotting times, and increased platelet reactivity (Fig. 3)^[27,28]^.

In aplastic anemia and related marrow failure syndromes, the coagulation profile is dominated by bleeding tendencies. Children frequently present with mucocutaneous hemorrhages, prolonged bleeding after minor trauma, and, in severe cases, disseminated intravascular coagulation (DIC). Laboratory evaluation typically reveals marked thrombocytopenia, prolonged prothrombin time (PT) and activated partial thromboplastin time (aPTT), and low fibrinogen levels, reflecting impaired hemostasis. Bleeding risk correlates directly with the severity of cytopenia and residual marrow function^[29,30]^. Pediatric ACD, often associated with chronic infections, autoimmune disorders, or malignancy, produces coagulation abnormalities largely mediated by systemic inflammation. Elevated cytokines such as interleukin-6 (IL-6) and tumor necrosis factor-alpha (TNF-α) promote tissue factor expression and thrombin generation. Clinically, affected children may experience mild bleeding or thrombotic events, with laboratory findings demonstrating elevated fibrinogen, increased D-dimer, and subtle platelet activation. The severity and type of coagulation disturbance often depend on the underlying disease and inflammatory burden (Table 1)^[31,32]^.

**Table 1:** Clinical Features and Coagulation Risk Profiles in Pediatric Anemia

Anemia Type	Key Clinical Features	Coagulation Abnormalities	Bleeding Risk	Thrombotic Risk	Notes/Clinical Considerations
Iron Deficiency Anemia (IDA)	Pallor, fatigue, growth delay, pica	Mild thrombocytosis or thrombocytopenia; impaired platelet aggregation	Low–moderate; usually mucocutaneous bleeding	Low	Severe or chronic IDA may prolong PT/aPTT; platelet function may be impaired, particularly in infants
Megaloblastic Anemia (Folate or B12 Deficiency)	Fatigue, pallor, neurologic symptoms (B12)	Thrombocytopenia, impaired platelet function	Moderate; epistaxis, easy bruising	Low	Correcting deficiency typically normalizes coagulation parameters
Sickle Cell Disease (SCD)	Pain crises, anemia-related pallor, splenomegaly	Platelet hyperactivity, elevated D-dimers, abnormal thrombin generation	Low–moderate; usually secondary to vaso-occlusive crises	High; risk of venous thromboembolism, stroke	Chronic hemolysis and endothelial dysfunction drive hypercoagulability
Beta-Thalassemia Major/Intermedia	Severe anemia, pallor, hepatosplenomegaly, growth delay	Abnormal platelet activation, elevated procoagulant microparticles, altered fibrinolysis	Low–moderate	High; particularly post-splenectomy or in older children	Splenectomy increases thrombotic risk; transfusion therapy affects coagulation profile
Anemia of Chronic Disease/Inflammation	Fatigue, pallor, underlying chronic illness	Mild thrombocytosis, subtle coagulation factor alterations	Low	Low–moderate; dependent on underlying disease	Risk depends on severity of inflammation and associated comorbidities
Acute Blood Loss/Hemorrhagic Anemia	Pallor, hypotension, tachycardia, shock in severe cases	Variable; dilutional coagulopathy in massive blood loss	High; risk proportional to volume lost	Low–moderate; dependent on resuscitation and transfusion	Immediate correction of volume and hemostatic support is critical

## Laboratory evaluation of coagulation abnormalities in anemia

Accurate laboratory evaluation is essential for identifying coagulation abnormalities in children with anemia and for guiding risk-based management strategies. Laboratory assessment provides insight into both bleeding and thrombotic tendencies, helping clinicians tailor interventions according to anemia type, severity, and individual patient risk factors (Tables 2 and 3). The evaluation can be broadly categorized into three tiers:

**Table 2:** Laboratory Evaluation of Coagulation Abnormalities by Pediatric Anemia Type

Anemia Type	Recommended Laboratory Tests	Clinical Indications/Rationale	Notes/Interpretation Tips
Iron Deficiency Anemia (IDA)	CBC (Hb, Hct, platelet count, MPV), Peripheral smear, PT, aPTT, Fibrinogen	Assess for thrombocytosis or thrombocytopenia; screen for subtle coagulation changes	PT/aPTT may be mildly prolonged in severe or chronic cases; platelet aggregation assays if bleeding is suspected
Megaloblastic Anemia (Folate/B12 Deficiency)	CBC, Peripheral smear, Reticulocyte count, PT, aPTT, Platelet function tests	Detect thrombocytopenia and platelet dysfunction; monitor for bleeding tendency	Correcting deficiency typically normalizes coagulation; platelet function tests useful in symptomatic patients
Sickle Cell Disease (SCD)	CBC, Reticulocyte count, D-dimer, Fibrinogen, PT, aPTT, Thrombin generation assays, Platelet function/activation markers	Evaluate hypercoagulability; monitor thrombotic risk, especially pre- and post-surgery	Elevated D-dimer and thrombin generation indicate ongoing coagulation activation; platelet activation markers correlate with vaso-occlusive events
Beta-Thalassemia Major/Intermedia	CBC, Reticulocyte count, PT, aPTT, Fibrinogen, D-dimer, Platelet function tests	Assess risk of thrombosis and bleeding, especially post-splenectomy	Transfusion history affects coagulation profile; frequent monitoring recommended in older children or post-surgical interventions
Anemia of Chronic Disease/Inflammation	CBC, ESR/CRP, PT, aPTT, Fibrinogen	Screen for mild coagulation alterations; monitor inflammatory contribution to hemostasis	Coagulation derangements usually mild; interpret alongside inflammatory markers
Acute Blood Loss/Hemorrhagic Anemia	CBC, PT, aPTT, Fibrinogen, Platelet count, Blood group & crossmatch	Immediate assessment for dilutional coagulopathy and bleeding risk	Rapid monitoring critical; guide transfusion and hemostatic support decisions

**Table 3:** Causes of False Positives/Negatives in Pediatric Coagulation Tests and Guidance for Managing Discordant Findings

Issue	Possible Causes	Laboratory Impact	Recommended Clinical Response
Pre-analytical Errors	Underfilled citrate tube, prolonged tourniquet use, hemolysis, delayed sample processing	*False elevation or reduction* in PT/aPTT; unreliable platelet function assays	Verify sample collection technique; repeat test; ensure correct fill volume and rapid processing
Age-Dependent Reference Ranges	Physiologic differences in neonatal and early childhood coagulation factor levels	*False abnormal* results when adult ranges are used	Reinterpret using age-adjusted reference intervals; consult pediatric hematology if values remain atypical
Sample Contamination	Heparin contamination from flushing lines; tissue factor contamination	*False prolongation* of aPTT or abnormal PT	Recollect sample from a clean venipuncture site; avoid drawing from lines unless fully flushed
Assay Variability	Differences in reagents, instrument sensitivity, or calibration	Inconsistent results between laboratories or assays	Review laboratory-specific reference ranges; consider repeating in the same facility for consistency
Platelet Count/Function Artifacts	Clotting in sample tube, EDTA-induced platelet clumping	*Falsely low* platelet count or impaired aggregation patterns	Repeat platelet count using citrate tube; examine peripheral smear for clumping
High Reticulocyte Counts in Hemolytic Anemia	Increased metabolic activity or circulating cell fragments	Spurious *false elevations* in coagulation markers (e.g., D-dimer)	Interpret results in context of hemolysis; repeat or confirm with alternative assays
Acute Illness or Fever	Transient inflammatory or consumptive changes	Temporary abnormal coagulation results	Reassess after acute illness resolves; correlate with clinical signs
Discordant Clinical-Laboratory Findings	Abnormal results with no bleeding signs, or bleeding symptoms with normal tests	Uncertain diagnostic direction	Repeat testing; review pre-analytical steps; use mixing studies; integrate family history, symptom scores, and anemia subtype; refer to specialist if persistent

### Basic screening tests

Initial assessment typically includes a complete blood count (CBC) with differential, platelet count, and standard coagulation tests such as prothrombin time (PT), activated partial thromboplastin time (aPTT), and fibrinogen levels. In iron deficiency anemia, reactive thrombocytosis and increased mean platelet volume (MPV) are frequently observed, whereas bone marrow failure syndromes may present with pronounced thrombocytopenia and prolonged clotting times. These basic tests provide a first-line understanding of hemostatic imbalance, though they have limitations in children. Pediatric reference ranges differ from adults and vary with age and developmental stage, making interpretation nuanced^[33,34]^.

### Advanced hemostatic assays

When standard screening indicates potential hemostatic disruption, specialized assays may be warranted. These include platelet function testing, thrombin generation assays, D-dimer, von Willebrand factor (vWF) antigen/activity, and soluble P-selectin measurements. In hemolytic anemias such as sickle cell disease and thalassemia, elevated D-dimer and soluble P-selectin reflect ongoing thrombin generation and endothelial activation, while NETosis-related markers, including circulating cell-free DNA and myeloperoxidase-DNA complexes, provide additional mechanistic insight into thromboinflammatory processes. These advanced assays, although informative, are not routinely available in all clinical settings and often require age-adjusted interpretation^[35–37]^.

### Genetic/thrombophilia panels

In select patients with recurrent thrombotic events, family history of thrombosis, or unusual laboratory patterns, genetic testing for inherited thrombophilias (e.g., Factor V Leiden, prothrombin G20210A mutation, antithrombin, protein C/S deficiencies) may be indicated. These tests assist in identifying children at heightened thrombotic risk and can guide long-term management strategies, including prophylactic anticoagulation when clinically justified^[38–40]^.

## Limitations in pediatric interpretation

Pediatric patients present unique challenges for laboratory evaluation. Hemostatic proteins, platelet function, and coagulation factor levels differ significantly from adults, particularly in neonates and infants. Age-specific reference ranges and dynamic developmental changes must be considered to avoid misinterpretation. Additionally, intercurrent illness, inflammation, or prior transfusions may alter results, emphasizing the need for integrated clinical-laboratory correlation^[41–43]^.

## Implications for risk assessment and management

Laboratory findings provide a critical framework for individualized risk stratification. In iron deficiency anemia, elevated platelet counts and MPV can signal increased thrombotic risk, while in hemolytic anemias, D-dimer, vWF, and NETosis markers identify children at heightened risk for stroke or venous thromboembolism. For bone marrow failure syndromes, prolonged PT/aPTT and thrombocytopenia guide bleeding prevention strategies. Integrating laboratory data with clinical assessment allows clinicians to prioritize interventions, including transfusions, iron therapy, anticoagulation, or close monitoring, while minimizing complications. Ultimately, structured laboratory evaluation enhances patient safety, informs preventive strategies, and supports evidence-based decision-making in pediatric anemia^[44,45]^.

## Implications for risk assessment and clinical management

Effective management of coagulation abnormalities in pediatric anemia requires a proactive, individualized approach that integrates clinical findings with laboratory insights. Risk assessment should consider the type and severity of anemia, laboratory markers of hemostatic imbalance, and the child’s comorbidities. For instance, children with hemolytic anemias such as sickle cell disease or thalassemia may benefit from targeted monitoring for thromboembolic events, whereas those with bone marrow failure syndromes require careful attention to bleeding risk^[46–48]^. Multidisciplinary collaboration between pediatricians, hematologists, and, when indicated, neurologists or critical care specialists is central to optimizing outcomes. Pediatricians provide longitudinal care, growth and nutritional support, and routine anemia management, while hematologists offer expertise in advanced diagnostics, interpretation of specialized coagulation assays, and guidance on therapeutic interventions, including transfusions or disease-modifying therapies. This team-based approach ensures that both bleeding and thrombotic risks are addressed in a coordinated, patient-centered manner^[49,50]^.

The decision to implement prophylactic anticoagulation must carefully balance potential benefits against the risks of bleeding, particularly in children with fluctuating platelet counts or coexisting hemostatic deficits. Current evidence supports individualized consideration, guided by laboratory findings such as elevated D-dimer, vWF, soluble P-selectin, or markers of NETosis, alongside clinical risk factors. In select high-risk cases, short-term anticoagulation may prevent thrombotic events, whereas in others, close monitoring and supportive care may be more appropriate^[51,52]^. Caregiver education represents a critical pillar of management. Parents and guardians should be counseled on recognizing early signs of bleeding or thrombosis, adhering to medication schedules, and ensuring timely follow-up for laboratory monitoring. Nutritional support, adherence to transfusion regimens, and awareness of triggers for vaso-occlusive crises in sickle cell disease further empower caregivers to participate actively in preventive strategies^[53,54]^. By integrating multidisciplinary care, evidence-based interventions, and caregiver engagement, clinicians can improve risk stratification, prevent adverse events, and optimize long-term outcomes in pediatric patients with anemia and coagulation abnormalities. This holistic approach ensures that laboratory findings translate into actionable clinical strategies tailored to the individual child’s needs^[55,56]^.

## Recommendations for clinical practice

To support clinicians in integrating coagulation assessment into the management of pediatric anemia, the following evidence-informed recommendations are proposed:

### Incorporate early coagulation screening in moderate-to-severe anemia

- Perform baseline PT, aPTT, fibrinogen, platelet count, and D-dimer testing in children with moderate–severe anemia or anemia with systemic symptoms.

Prioritize screening in conditions with known prothrombotic risk such as sickle cell disease and thalassemia.

### Use age-adjusted reference ranges for interpretation

- Interpret coagulation parameters using pediatric- and age-specific reference intervals to avoid misclassification.

Engage pediatric laboratory services or hematologists when local reference ranges are limited.

### Repeat and verify abnormal results before management decisions

- Reassess any abnormal coagulation test – especially if discordant with clinical findings – to rule out pre-analytical errors.
- Confirm tube fill volume, sample handling, and timing of processing before attributing abnormalities to pathology.

### Integrate clinical symptoms with laboratory findings for risk stratification

- Combine bleeding symptoms, thrombosis history, family history, and anemia subtype with laboratory data to categorize patients into low-, intermediate-, or high-risk groups.

Use simple scoring tools (e.g., pediatric bleeding assessment tools) to reinforce risk assessment.

## Monitor high-risk groups more frequently

- Children with sickle cell disease, transfusion-dependent thalassemia, inflammatory anemia, or recurrent infections should undergo periodic coagulation monitoring due to dynamic risk profiles.

Increase monitoring frequency during acute illness, dehydration, infection, or vaso-occlusive pain crises.

### Apply a structured approach to discordant findings

When laboratory values and clinical presentation do not align, follow a stepwise process:

(a) Verify sample quality

(b) Repeat the test

(c) Consider mixing studies

(d) Align results with symptoms and anemia etiology

(e) Refer to pediatric hematology for persistent discrepancies

### Educate caregivers and patients on warning signs

- Provide clear guidance on red-flag symptoms such as unexplained bruising, recurrent epistaxis, limb swelling, prolonged fever, or sudden neurologic changes.

Use simple checklists to support early recognition and prompt care-seeking.

### Use multidisciplinary management for complex cases

- Engage pediatric hematologists, transfusion medicine specialists, and laboratory scientists when evaluating ambiguous or high-risk coagulation profiles.
- Coordinate with nutritionists, infectious disease experts, and cardiologists for anemia cases with overlapping systemic risk.

### Document and communicate risk category at every visit

Clearly record each child’s coagulation risk category in the medical record.

Update the risk category as anemia severity, clinical status, or laboratory findings change.

### Advocate for strengthened laboratory capacity in resource-limited settings

- Support initiatives aimed at improving pediatric reference ranges, point-of-care coagulation testing, and standardized training for pre-analytical procedures.
- Encourage the establishment of regional pediatric hematology networks to improve diagnostic and management consistency.

## Conclusion

Coagulation abnormalities represent a significant but often overlooked contributor to morbidity in pediatric anemia, with prevalence and severity varying across anemia subtypes and influenced by underlying pathophysiologic mechanisms. Effective risk assessment requires integrating clinical features with targeted laboratory markers, applying age-appropriate reference ranges, and recognizing the potential for false or discordant results that may complicate interpretation. Moving forward, improved longitudinal studies, standardized pediatric-specific laboratory algorithms, and expanded research on high-risk groups such as sickle cell disease and thalassemia are essential to refine diagnostic accuracy and strengthen early intervention strategies. By aligning clinical vigilance with evidence-based assessment tools, healthcare providers can enhance risk stratification and deliver more timely, individualized care for children with anemia.
